# Draft Genome Sequences of Six Strains of Lactococcus lactis (Phylum *Firmicutes*), Spanning the Seeds of *Cucumis sativus* L. (Cucumber), *Cucumis melo* L. (Cantaloupe), and *Cucurbita pepo* var. *turbinate* (Acorn Squash)

**DOI:** 10.1128/MRA.00665-20

**Published:** 2020-09-10

**Authors:** Eman M. Khalaf, Manish N. Raizada

**Affiliations:** a Department of Plant Agriculture, University of Guelph, Guelph, Ontario, Canada; b Department of Microbiology and Immunology, Faculty of Pharmacy, Damanhour University, Damanhour, Egypt; Georgia Institute of Technology

## Abstract

We announce the draft genome sequences of six strains of Lactococcus lactis (EKM101L, EKM102L, EKM201L, EKM203L, EKM501L, and EKM502L). These candidate plant probiotics were isolated from surface-sterilized seeds of *Cucumis sativus* L. (cucumber), *Cucumis melo* L. (cantaloupe), and *Cucurbita pepo* var*. turbinate* (acorn squash). They display beneficial activities, including biocontrol.

## ANNOUNCEMENT

Lactic acid bacteria produce diverse antimicrobial peptides, leading to their extensive use in the food industry, in crop production, and as human probiotics (1, 2). *Lactococcus* is a prevalent genus of the cucurbit seed microbiome (3). *Lactococcus* comprises 11 species, including Lactococcus lactis, a safe and important industrial bacterial species (4). Seeds of Cucumis sativus L. (cucumber), Cucumis melo L. (cantaloupe), and Cucurbita pepo var. *turbinate* (acorn squash) were surface sterilized and then gently ground in 50 mM Na_2_HPO_4_ buffer using autoclaved mortars. To isolate seed-associated endophytes, the ground seed suspensions were cultured by streaking onto peptone-dextrose agar (PDA), Reasoner's 2A (R2A) agar, and LGI agar (5) plates and then incubated for up to 7 days at 28°C (6). Six strains of L. lactis (EKM101L, EKM102L, EKM201L, EKM203L, EKM501L, and EKM502L) were isolated and identified using the 16S rRNA universal primer pair 799F/1492R, and the gene sequences were deposited in GenBank (accession numbers KT281324, KT281446, KT281325, KT281327, KT281328, and KT281329, respectively) (6). *In vitro* characterization revealed the ability of all these strains to solubilize mineral phosphate and to suppress oomycetes (*Phytophthora capsici* and *Pythium aphanidermatum*) (6, 7). Concerning additional tested traits, only EKM102L and EKM501L produced auxin and RNase, while EKM101L produced siderophores, acetoin, and RNase (6, 7). Furthermore, only EKM203L, EKM501L, and EKM502L reduced the disease index of the foliar fungal pathogen *Podosphaera fuliginea* (cucumber powdery mildew) *in planta* (7).

From −80°C original glycerol stocks, strains were cultured on LB agar, and single colonies were incubated overnight in LB broth at 37°C at 250 rpm. Genomic DNA was extracted from pellets using the DNeasy UltraClean microbial kit (product number 12224-50; Qiagen) and then adjusted to 50 ng/μl. Libraries were prepared using the TruSeq DNA Nano library preparation kit (KAPA HyperPrep kit, product number KK8504) and sequenced using an Illumina NovaSeq 6000 system, generating 1,726,647 (EKM101L), 1,697,818 (EKM102L), 1,710,169 (EKM201L), 1,689,628 (EKM203L), 1,808,657 (EKM5101L), and 1,715,177 (EKM502L) raw reads in the 150-bp paired-end format. Using the EvoCAT (Evogene Clustering and Assembly Toolbox) pipeline, raw reads were filtered (Phred quality score of 30), *de novo* assembled, and then taxonomically identified using KmerFinder v3.1 (8), which resulted in 180-fold (EKM101L), 181-fold (EKM102L), 175-fold (EKM201L), 179-fold (EKM203L), 191-fold (EKM5101L), and 181-fold (EKM502L) sequence coverage, compared to L. lactis subsp*. lactis* strain S0 (GenBank accession number CP010050.1), with query coverage of 72.83% to 73.17%. The genome annotation was performed using the NCBI Prokaryotic Genome Annotation Pipeline (PGAP) v4.12 (9). Default parameters were used for all software unless otherwise specified. Assembly metrics and annotated features are shown in Table 1.

**TABLE 1:** Genome features and accession numbers of *Lactococcus* strains isolated from cucurbit seeds

Isolate	Bacterial species^*a*^	Genome size (bp)	No. of contigs	*N*_50_ (bp)	Total no. of predicted genes	No. of protein-coding genes	G+C content (%)	SRA accession no.	GenBank accession no.
EKM101L	*L. lactis* subsp*. lactis*	2,711,771	98	234,846	2,855	2,707	35	SRR11053546	JAALFN000000000
EKM102L	*L. lactis* subsp*. lactis*	2,707,623	84	217,917	2,839	2,694	35	SRR11053525	JAALFS000000000
EKM201L	*L. lactis* subsp*. lactis*	2,709,521	94	485,885	2,852	2,704	35	SRR11051663	JAALEP000000000
EKM203L	*L. lactis* subsp*. lactis*	2,705,547	77	487,028	2,820	2,680	35	SRR11051661	JAALEQ000000000
EKM501L	*L. lactis* subsp*. lactis*	2,708,771	86	234,846	2,841	2,694	35	SRR11043519	JAALFI000000000
EKM502L	*L. lactis* subsp*. lactis*	2,699,645	111	391,527	2,856	2,711	35	SRR11043894	JAALFJ000000000

a Strain taxonomic identification according to the updated GenBank databases.

From the annotated genomes, we detected coding regions predicted to underlie the previously identified beneficial traits, namely, phosphatase PAP2 family protein (phosphate solubilization) (10), bacteriocins (11), flavin reductase family protein (H_2_O_2_ production) (12), phosphoketolase family protein (lactic acid production) (13, 14), gallidermin/nisin family lantibiotic (11), serine protease (15, 16), and chitinases (17, 18). Additional genes were detected in all strains despite the corresponding activities not being consistently expressed (6, 7), including those encoding 2,3-butanediol dehydrogenase (acetoin production) (19) and indole-3-glycerol phosphate synthase TrpC (auxin/indole-3-acetic acid production) (20, 21). Interestingly, ferrous iron (Fe^2+^) transport proteins A and/or B were identified; these are major components of the Feo system for the acquisition of Fe^2+^, which is the abundant form of iron under anaerobic conditions or low pH (22). These findings reveal the genetic reservoir of L. lactis, consistent with its diverse use in the food industry and as plant and human probiotics.

### Data availability.

This whole-genome shotgun project has been deposited in DDBJ/EMBL/GenBank, along with raw Illumina reads in the SRA, at the accession numbers noted in Table 1.
